# Liver resection in a patient with persistent positive PCR test for coronavirus disease 2019 (COVID-19): a case report

**DOI:** 10.1186/s40792-022-01553-z

**Published:** 2022-10-20

**Authors:** Akiho Sugita, Fuyuki F. Inagaki, Nobuyuki Takemura, Mai Nakamura, Kyoji Ito, Fuminori Mihara, Kei Yamamoto, Shinichiro Morioka, Norihiro Kokudo

**Affiliations:** 1 Department of Surgery, National Center for Global Health and Medicine, Hepato-Biliary-Pancreatic Surgery Division, 1-21-1 Toyama, Shinjyuku-ku, Tokyo, 162-8655 Japan; 2 National Center for Global Health and Medicine, Disease Control and Prevention Center, Tokyo, Japan

**Keywords:** Coronavirus disease 2019 (COVID-19), Severe acute respiratory syndrome coronavirus 2 (SARS-CoV-2), Surgery, Polymerase chain reaction (PCR), Hepatocellular carcinoma

## Abstract

**Background:**

The perioperative mortality rate is high in patients with coronavirus disease 2019 (COVID-19), and infection control measures for medical care providers must be considered. Therefore, the timing for surgery in patients recovering from COVID-19 is difficult.

**Case presentation:**

A 65-year-old man was admitted to a hospital with a diagnosis of moderate COVID-19. He was transferred to our hospital because of risk factors, including heavy smoking history, type 2 diabetes mellitus, and obesity (BMI 34). Vital signs on admission were a temperature of 36.1 °C, oxygen saturation > 95% at rest, and 94% on exertion with 3 L/min of oxygen. Chest computed tomography (CT) showed bilateral ground-glass opacities, predominantly in the lower lungs. Contrast-enhanced abdominal CT incidentally revealed a liver tumor with a diameter of 80 mm adjacent to the middle hepatic vein, which was diagnosed as hepatocellular carcinoma (HCC). After being administered baricitinib, remdesivir, dexamethasone, and heparin, the patient’s COVID-19 pneumonia improved, his oxygen demand resolved, and he was discharged on day 13. Furthermore, the patient was initially scheduled for hepatectomy 8 weeks after the onset of COVID-19 following a discussion with the infection control team. However, 8 weeks after the onset of illness, a polymerase chain reaction (PCR) test was performed on nasopharyngeal swab fluid, which was observed to be positive. The positive results persisted till 10 and 11 weeks after onset. Both Ct values were high (≥ 31) out of 45 cycles, with no subjective symptoms. Since we determined that he was no longer contagious, surgery was performed 12 weeks after the onset of COVID-19. Notably, medical staff wearing personal protective equipment performed extended anatomical resection of the liver segment 8 ventral area in a negative-pressure room. The patient had a good postoperative course, with no major complications, including respiratory complications, and was discharged on postoperative day 14. Finally, none of the staff members was infected with COVID-19.

**Conclusions:**

We reported a case regarding the timing of surgery on a patient with persistently positive PCR test results after COVID-19, along with a literature review.

## Background

As of October 11, 2021, over 237 million confirmed coronavirus disease 2019 (COVID-19) cases and 4.8 million deaths had been reported, according to the WHO dashboard [1]. In Japan, over 1.7 million have been diagnosed with COVID-19, and the pandemic has claimed over 17 thousand lives [1].

During the COVID-19 pandemic, most facilities experienced limited use of medical resources. The European Association for the Study of the Liver (EASL) Position Paper and the Working Group report of the Japan Association of Molecular Targeted Therapy for Hepatocellular Carcinoma (JAMTT–HCC) recommended postponement of local therapy, including surgery for patients with hepatocellular carcinoma during the COVID-19 pandemic [2, 3]. According to the survey conducted in eight hospitals from eight countries, including our hospital, treatment of patients without COVID-19 decreased by 0–70% depending on the extent of the epidemic [4]. In addition, another survey revealed that 10 out of 17 hospitals in Japan had limited elective surgeries during the waves of COVID-19, and some non-essential surgeries were postponed or canceled [5].

Morris et al. reported a mean preoperative COVID-19 positive test rate of 0.74% (18 of 2437 patients) in adult patients [6]. Although COVID-19 preoperative positive test results differ from region to region depending on the prevalence of COVID-19 in the community, it is imperative to identify patients with severe acute respiratory syndrome coronavirus 2 (SARS-CoV-2) infection, so that surgery can be safely postponed. This process protects not only the patient but also the healthcare worker by avoiding unnecessary exposure to patients infected with SARS-CoV-2. Surgical patients have an increased risk of perioperative morbidity and mortality regardless of their symptoms [7]. Doglietto et al. showed that the 30-day risk of mortality, the odds of perioperative pulmonary complications, and the odds of thrombotic complications were higher in patients with COVID-19 [8].

This report demonstrated the case of a 65-year-old male who underwent extended anatomical resection of the ventral area of liver segment 8 for hepatocellular carcinoma, despite persistent positive polymerase chain reaction (PCR) results for SARS-CoV-2.

## Case presentation

A 65-year-old male with a 3-day history of fever and dullness was admitted to a local hospital for pneumonia. Subsequently, he was diagnosed with COVID-19, which was confirmed by a positive PCR test result using a nasopharyngeal swab. Therefore, his previous doctor started treating him by administering baricitinib (4 mg oral), remdesivir (200 mg IV), dexamethasone (6 mg oral), and subcutaneous heparin 10,000 U. However, he was transferred to our hospital the next day because of potential risk factors for the severity of COVID-19 as follows: increasing age (65 years), past heavy smoking (50 cigarettes per day for 26 years), type 2 diabetes mellitus, and obesity (body mass index = 34.2). Physical examination upon admission to our hospital revealed a body temperature (BT) of 36.1 °C, blood pressure (BP) of 133/65 mmHg, heart rate (HR) of 72 beats/min, and a Glasgow Coma Scale of E4V5M6. His oxygen saturation (SpO_2_) was > 95% on room air; however, it easily dropped below 95% with low-level exertion, and he required an oxygen flow rate of 3 L/min to maintain 94%. Furthermore, the laboratory results were as follows: white blood cell count (WBC) of 2250/μl, C-reactive protein (CRP) of 2.18 mg/dL, D-dimer of 1.4 μg/mL, activated partial thromboplastin time (APTT) of 33 s, prothrombin time-international normalized ratio (PT-INR) of 1.05, and hemoglobin A1c(HbA1c) of 6.6%.

**Fig. 1:**
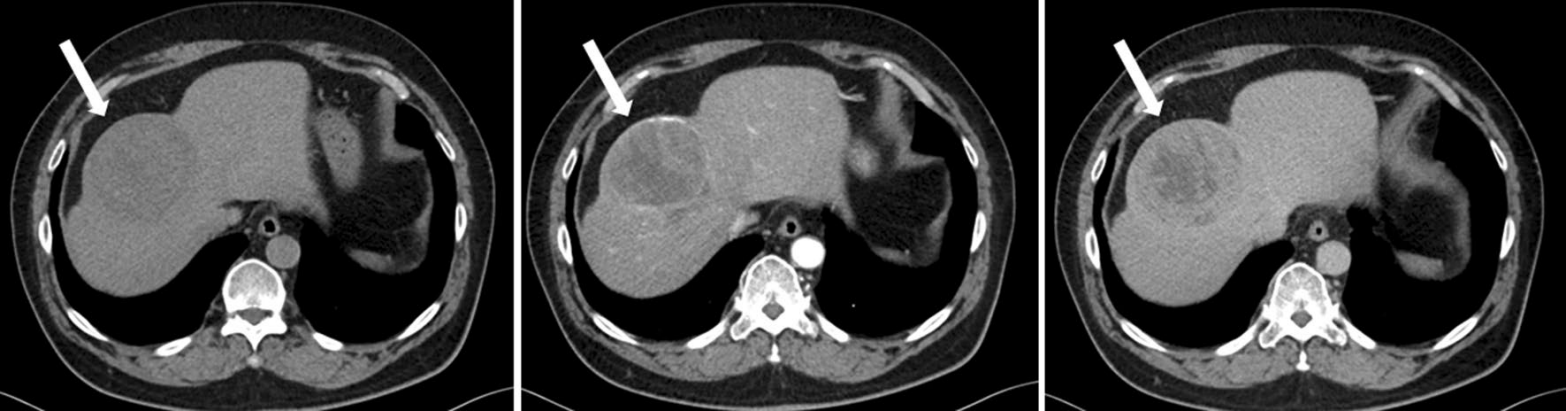
Abdominal computed tomography (CT) images. Contrast-enhanced computed tomography revealed a tumor in segment 8 of the liver. The tumor was 80 mm in diameter and was adjacent to the middle hepatic vein. The white arrow indicates the tumor. Left: plain; middle: arterial phase; right: portal or delayed phase

**Fig. 2:**
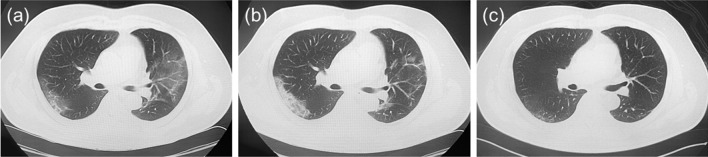
Chest computed tomography (CT) images. **a** CT image 5 days after onset of symptoms. Bilateral ground-glass opacities (GGOs) were observed with lower-lung predominance. **b** CT image taken 11 days after onset, showing slightly elevated CT values of GGOs. **c** CT image taken 54 days after onset, showing the disappearance of GGOs

Computed tomography (CT) revealed bilateral ground-glass opacities with lower lung predominance and also showed a tumor 80 mm in diameter in segment 8 of the liver (Figs. 1 and 2). The tumor margin was well-enhanced in the arterial phase and washed out in the delayed phase, suggesting hepatocellular carcinoma (HCC). The tumor was adjacent to the middle hepatic vein (MHV), and the main feeder was the P8 ventral branch. No other nodules suggestive of HCC were identified. There was no evidence of lymph node swelling or metastasis to the other organs. While the serum levels of protein induced by vitamin K antagonist-II (PIVKA-II) were 346 mAU/mL (reference range: < 40 mAU/mL), other tumor markers, including α-fetoprotein (AFP), carcinoembryonic antigen (CEA), and carbohydrate antigen 19-9 (CA19-9), were all within the reference ranges, the hepatitis virus test results were positive for hepatitis C virus (HCV) infection and preexisting hepatitis B virus infection.

Although he was classified as a moderate patient with COVID-19, he responded well to treatment. Baricitinib was discontinued on admission, because he was an HCV carrier, and remdesivir was withdrawn on day 7 from the date of COVID-19 onset. In addition, subcutaneous heparin was administered until day 9, and dexamethasone was tapered from 6 to 1 mg. Given the good clinical course, the patient was discharged from the hospital on day 13 of the onset of COVID-19. After consultation with the disease control and prevention center in our hospital, surgery for HCC was scheduled 8 weeks after the onset of COVID-19. Eight weeks after onset, a PCR test using a nasopharyngeal swab showed a positive result (N 501 mutation-positive, E484K mutation-negative). Subsequently, we re-examined the PCR test 10 weeks after onset, and the result remained positive. This result was obtained using the Cobas SARS-CoV-2 detection kit (Roche Diagnostics K.K., Japan), and the cycle threshold (Ct) value was 36.7 out of 45 cycles. A PCR test was conducted again after a week, and the Ct value was 31.7 out of 45 cycles. The patient was no longer considered contagious given the fact that he was asymptomatic and his last Ct value was relatively high. During the waiting period before surgery, tumor marker values and tumor size remained virtually unchanged. Because there were no symptoms suspicious of impending rupture and no rapid increase in tumor size, non-surgical options such as transarterial chemoembolization (TACE) or chemotherapy were not suggested during the waiting period.

Consequently, we decided to perform the surgery 12 weeks after the onset of COVID-19. The preoperative liver function test results were as follows: total bilirubin, 1.0 mg/dL; albumin, 3.9 g/dL; prothrombin test, 81%; indocyanine green retention rate at 15 min, 24.5%. The liver damage score was ranked as class A, and the Child–Pugh class was A with 5 points.

**Fig. 3:**
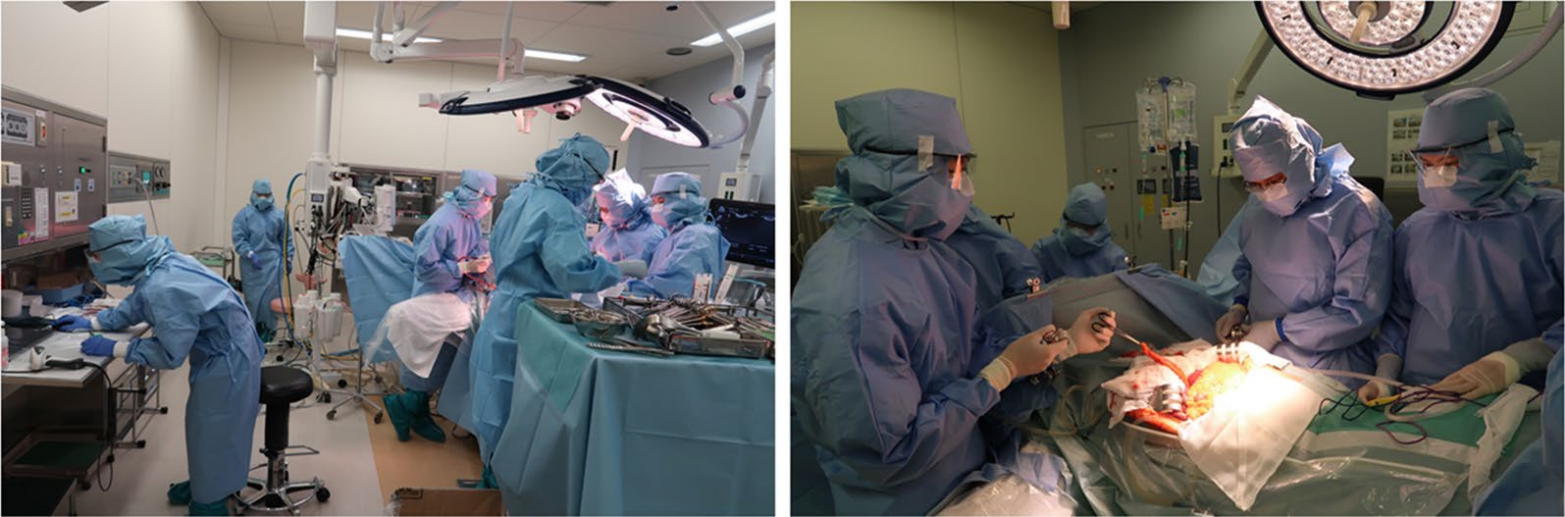
View of the operating room. The operation was performed in a negative pressure room. All medical staff wore personal protective equipment, including N95 respirators

**Fig. 4:**
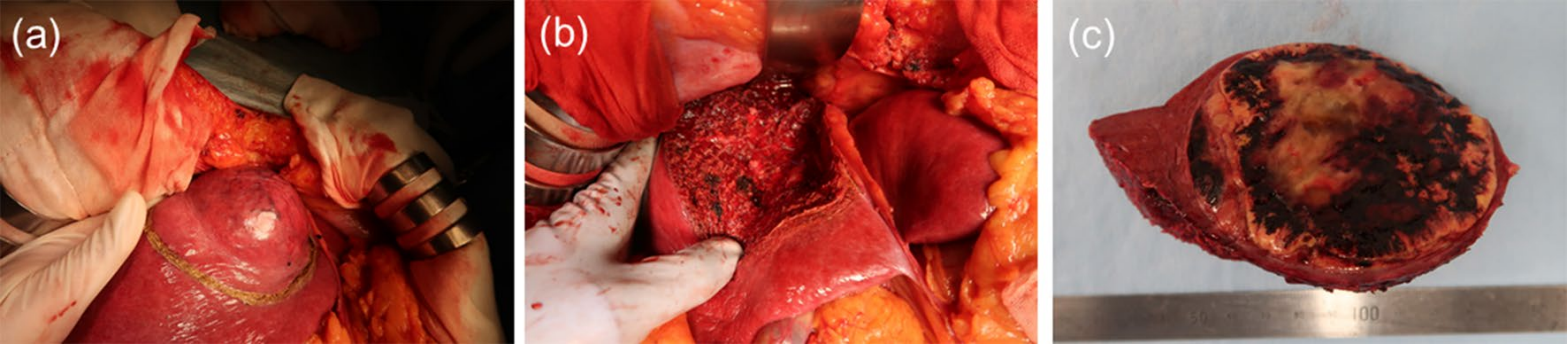
Intraoperative photographs and macroscopic view of the resected specimen. **a** Tumor was located in segment 8 of the liver. The tumor protruded from the liver surface. **b** Resected surface of the liver after extended anatomical resection of the liver segment 8 ventral area. **c** Macroscopic view of the resected specimen. A yellowish, solid 7 cm tumor was seen, with hemorrhage and necrosis

Ninety days after the onset of COVID-19, extended anatomical resection of the liver segment 8 ventral area was performed in a negative-pressure room, and all surgical staff wore personal protective equipment, including N95 masks (Fig. 3). To reduce aerosol exposure to medical staffs, only a minimal number of personnel remained in the operating room during intubation and extubation. Surgical smoke evacuators were not used, because there was no evidence at the time of surgery that COVID-19 was transmitted by surgical smoke. The operative time was 225 min, and the estimated blood loss was 1061 mL. The resected specimen showed a yellowish tumor with hemorrhage and necrosis (Fig. 4). In addition, pathological examination revealed well-to-moderately differentiated HCC (75 × 70 × 65 mm in size, simple nodular type, e.g., fc(+), fc-inf(−), sf(+), S0, Vp0, Vv0, Va0, B0, pT2N0M0, pStage II). Notably, the postoperative course was uneventful, and the patient was discharged on the 14th postoperative day. Unlike the operating room, where the risk of aerosol exposure is high, the infection control team determined that the infectivity was low, so the usual standard precautions were taken in the wards, including mutual wearing of surgical masks. During the hospital stay, the patient had no COVID-19 symptoms, and no secondary infection by healthcare workers was observed.

## Discussion

In this study, we reported a case of a patient with COVID-19 and HCC whose test results for SARS-CoV-2 remained positive 11 weeks after the onset of COVID-19. At our institution, in consultation with the infection control team, we usually perform the surgery on patients with COVID-19 8 weeks after the onset of COVID-19, which we believe is a sufficient time interval. Briefly, possible reasons for positive tests in recovered patients with COVID-19 include false-positive PCR tests, reinfection with SARS-CoV-2, and detection of dead SARS-CoV-2. However, in this study, false positives were unlikely in the patient, because the repeated PCR tests were all positive.

Reinfection cannot be denied in this patient, but it is less probable based on the fact that relatively few cases of reinfection in short intervals (≦ 3 months) have been reported [9, 10]. It is difficult to determine whether this phenomenon is simply due to prolonged viral shedding or prolonged infection. In our institution, we sometimes repeat PCR tests to check the trend of the Ct values. The distinction between prolonged viral shedding and prolonged infection is vital for two reasons, which are explained below.

First, we have to be fully careful regarding the potential risk of nosocomial infection transmission during surgery under general anesthesia. PCR tests for SARS-CoV-2 are more sensitive and specific than antigen tests. In Japan, several types of COVID-19 PCR tests are available, including SmartGene (MIZUHO MEDY Co., Ltd, Japan), FilmArray (BioFire Diagnostics, Salt Lake City, UT, USA), GeneXpert (Cepheid, Sunnyvale, CA, USA), ID NOW (Abbott, Scarborough Diagnostics) [11], and Cobas SARS-CoV-2 detection kit (Roche Diagnostics K.K., Japan). FilmArray does not show the Ct value and cannot estimate the viral load [11], whereas other tests, including the SmartGene and Cobas assays, display an alternative number of cycles. For this patient, the FilmArray test confirmed SARS-CoV-2 infection on his first admission, revealing that his SARS-CoV-2 had the N501Y mutation, which has been linked to increased transmission in SARS-CoV-2 variants found in several strains from the UK, South Africa, and Brazil [12]. In addition, Cobas assay (ORF1 a/b and E gene target [13]) was used for recheck tests for this patient on days 72 and 81 of COVID-19 to estimate the viral load. Di Tian et al. surveyed Ct values among 7,440 undergraduate students at Tulane University in New Orleans [14]. Although they concluded that Ct values at the individual level could not predict transmissibility, they observed a trend that the symptomatic groups (spreaders and non-spreaders) and the spreader groups (with or without symptoms) tended to include more individuals with lower Ct values (< 24). La Scola et al. investigated 183 samples that tested positive using reverse transcription (RT-PCR) targeting the E gene and concluded that Ct values above 33–34 utilizing their RT-PCR system are not contagious [15]. Jaafar et al. evaluated 1941 quantitative PCR samples and positive cell cultures of SARS-CoV-2 and suggested that when the PCR is positive beyond 10 days, the Ct value is often greater than 30, which these rare cases should not impact public health decisions [16].

From the viewpoint of epidemiology, mathematical modeling, and virology, the infectious period of COVID-19 is considered to be 7–12 days maximum from the onset of the disease. The secondary infection rate was zero among 852 contacts whose initial exposure to the index case occurred after day 6 [17]. Based on a mathematical model for infectiousness, the basic reproductive number R0 = 2.0 in the early stages of the epidemic in China (46% from presymptomatic individuals, 38% from symptomatic individuals, 10% from asymptomatic individuals, and 6% from environmentally mediated transmission); however, the estimated reproductive number is nearly zero 12 days after infection [18]. According to a survey of upper respiratory tract samples from 176 symptomatic cases, the median duration of virus shedding measured by culture was 4 days, and the culture-positive rate was significantly higher in the first week than in the second week [19].

Prolonged infectivity has been observed in immunocompromised patients. It has been reported that the recovery of infectious virus for approximately 70 days after the first positive result has been reported in a subset of immunocompromised patients (e.g., HIV diseases, chronic kidney disease, transplant status, hepatic fibrosis, and hepatic failure); however, patients with liver cancer without transplantation were not included in this study [20]. It has been reported that resolution with a Ct value > 30 is longer in patients with hematologic cancer than in patients with solid tumors [21], and prolonged infection background is supposed to be mainly in patients with severe liquid immunodeficiency (e.g., hematologic malignancies, rituximab users) [22]. Therefore, although this patient remained PCR-positive even after 12 weeks, there is low possibility of isolation of the infectious virus.

Second, the prognosis may not be good if surgery is performed, while the disease has not firmly improved. Reportedly, patients with cancer have a higher risk of severe events of SARS-CoV-2 [23] and perioperative SARS-CoV-2 infections, which are known to lead to high mortality rates [7, 8, 24, 25]. Nepogodiev et al. conducted an observational cohort study on patients with SARS-CoV-2 infection who underwent surgery at 235 hospitals in 24 countries and reported that 30-day mortality was 21.1% (62 of 294) in patients who were confirmed with SARS-CoV-2 infection 7 days before the operation [24]. Therefore, surgery for this patient was postponed until COVID-19 was cured.

Reportedly, perioperative SARS-CoV-2 infection is associated with an increased risk of postoperative complications and mortality [7, 8, 24, 25]; however, the association has not yet been fully identified. Therefore, the timing for surgery remains under discussion. Besides, it has been reported that patients with cancer and prolonged SARS-CoV-2 RNA detection (defined by positive RT-qPCR duration ≥ 40 days) showed typical immunopathology (e.g., the prolonged systemic release of type 1 IFN), which might be the immunological hallmark of severe COVID-19 [26]. Consequently, we needed careful observation when the test results remained positive 8 weeks after the onset of COVID-19. However, a prospective cohort study of 140,231 patients in 116 countries showed that the risks of 30-day postoperative mortality and 30-day postoperative pulmonary complications decreased to baseline in patients who underwent surgery ≥ 7 weeks after SARS-CoV-2 diagnosis [25]. Ct values of the test results for this patient remained above 30; therefore, the risks of mortality and complications related to both surgery and COVID-19 itself were supposed to be relatively low.

Cancer spreading, chemotherapies, and virus-induced lymphopenia probably affect viral clearance, as some types of viruses (e.g., influenza, parainfluenza, rhinovirus, and seasonal coronavirus) have been reported to be related to prolonged viral shedding [26]. Considering that the patient was diagnosed with HCC, it is understandable that the viral excretion persisted. Since a test-based approach in all immunocompromised patients may lead to prolonged isolation. In a case series of immunocompromised patients, unlike patients with hematologic tumors, none of the solid organ transplant patients had positive viral cultures, and the mean Ct value of negative viral cultures was 20.5 [27]. It has been reported that viral cultures do not test positive unless the amount of viral nucleic acid is high (viral loads > 7 log10 RNA copies/mL) [28].

In addition to the low possibility of prolonged infection considering the patient’s immunologic status [22, 29], as in the case of prolonged infection, the viral load remains low, and ongoing lung inflammation begins to appear [29–31]. The risk of prolonged infection was judged to be low considering the patient’s background and the fact that the patient's Ct value transitioned around 30–35, which suggests a low viral value, and the patient was asymptomatic. Therefore, it was decided that there was no need to postpone the surgery. Although the patient in this case had persistent PCR positivity, the results of Ct values were useful for the assessment of infectivity and COVID-19 disease status. Ct values provided the basis for the decision on the criteria for ending isolation and precautions for COVID-19, and when to perform the surgery.

## Conclusions

We reported the case of a 66-year-old male who underwent hepatectomy for HCC, who had been diagnosed with COVID-19 12 weeks before surgery, and his COVID-19 PCR test results remained positive at the time of surgery. However, given that the patient was asymptomatic and his last Ct value was relatively high (> 30), we concluded that he was unlikely contagious. Consequently, we performed the operation, and the patient was discharged from the hospital without any postoperative complications or secondary infections to the medical staff. Summarily, the timing of surgery for a patient who has recovered from COVID-19 should be carefully determined based on the patient’s current condition and the assessment of transmissibility.

## Data Availability

The data sets analyzed in the current study are not publicly available, because they contain information that may compromise the privacy of the patient but are available from the corresponding author on reasonable request.

## Abbreviations

PCR: Polymerase chain reaction
HCV: Hepatitis C virus
JAMTT–HCC: Japan Association of Molecular Targeted Therapy for Hepatocellular Carcinoma
EASL: European Association for the Study of the Liver
SARS-CoV-2: Severe acute respiratory syndrome coronavirus 2
CT: Computed tomography
COVID-19: Coronavirus disease 2019
MHV: Middle hepatic vein
PIVKA-II: Vitamin K antagonist-II
AFP: α-Fetoprotein
CEA: Carcinoembryonic antigen
CA19-9: Carbohydrate antigen 19-9
GGOs: Ground-glass opacities
